# AXL in myeloid malignancies – an elusive target?

**DOI:** 10.1186/s40364-024-00704-8

**Published:** 2024-12-18

**Authors:** Pia Aehnlich, Katharina Leuchte, Claudia Schöllkopf, Sara Fresnillo Salo, Tina J. Seremet, Estrid Høgdall, Özcan Met, Kirsten Grønbaek, Per thor Straten

**Affiliations:** 1 Department of Oncology, National Center for Cancer Immune Therapy (CCIT-DK), Copenhagen University Hospital Herlev, Herlev, Denmark; 2 Department of Hematology, Copenhagen University Hospital, Copenhagen, Denmark; 3 Department of Pathology, Copenhagen University Hospital Herlev, Herlev, Denmark; 4 Biotech Research and Innovation Center (BRIC), University of Copenhagen, Copenhagen, Denmark; 5 Department of Clinical Medicine, Faculty of Health and Medical Sciences, University of Copenhagen, Copenhagen, Denmark

**Keywords:** AXL, Acute myeloid leukemia (AML), Tumor immunology, TAM receptors

## Abstract

The TAM receptor tyrosine kinase family member AXL plays critical roles in tissue homeostasis, survival, chemoresistance, and motility. This study investigates the receptor expression in six AML cell lines and bone marrow myeloblasts from 25 patients with myeloid neoplasms. We found that AXL expression was generally absent or very low in AML myeloblasts. These findings suggest that the efficacy of AXL inhibitors may not depend on AXL positivity but rather on alternative therapeutic mechanisms, such as inducing significant immune responses.

**Supplementary Information:**

The online version contains supplementary material available at 10.1186/s40364-024-00704-8.

To the editor,

The TAM receptor tyrosine kinase family—Tyro3, AXL, and MerTK—plays key roles in regulating adult tissue homeostasis. Abnormal AXL signaling is linked to tumor-promoting and anti-apoptotic properties, including epithelial-to-mesenchymal transition (EMT), angiogenesis, cell survival, and therapy resistance [1]. AXL was discovered in 1991 in two chronic myeloid leukemia (CML) patients [2], and its expression is detected in various cancers [3, 4]. Due to AXL’s role in hematopoiesis, research has focused on acute myeloid leukemia (AML), characterized by clonal expansion of undifferentiated myeloid precursor cells. Despite advances in understanding AML’s genetic and epigenetic alterations, treatment has barely changed, with poor survival rates highlighting the need for new therapies.

AXL has been shown as overexpressed in leukemic cells, with over 50% of AML patients showing AXL mRNA expression [5]. AXL protein in de novo AML patients is an independent risk factor for poor prognosis, decreasing chemotherapy response rates [5, 6]. AXL knockout and pharmacologic inhibition restore sensitivity to cytotoxic therapies [3, 7]. This underscores the translational potential of the GAS6-AXL pathway. Consequently, AXL-targeted therapies are under clinical investigation, with some already been approved by EMA and FDA [3].

In this study, we assessed Tyro3, AXL and MerTK protein and surface expression in 6 AML cell lines and bone marrow-derived myeloblasts from 25 patients with myeloid neoplasms (22 AML, 2 CML in blast crisis and 1 CMML transformed into AML). The study was performed in accordance with the Declaration of Helsinki, and all 25 samples were collected following written informed consent. All patients were above 18 years of age. Capital Region’s Ethics Committee approval H-20,046,888. Samples were stored in the Danish CancerBiobank. Epidemiologic and clinical characteristics of the included patients are summarized in Supplementary Table 1.

We investigated the surface expression of Tyro3, AXL and MerTK in AML cell lines (Mono-Mac-1,

KG-1, OCI-AML2, OCI-AML3, HL-60, THP-1) using flow cytometry. MerTK was expressed on several cell lines, Tyro3 on one, but none showed AXL expression (Fig. 1A-B). AXL protein was detected only in two dim samples (Fig. 1C). To confirm the absence of AXL expression, we validated AXL antibody specificity for flow cytometry and Western blot (Supplementary Fig. S1).

**Fig. 1:**
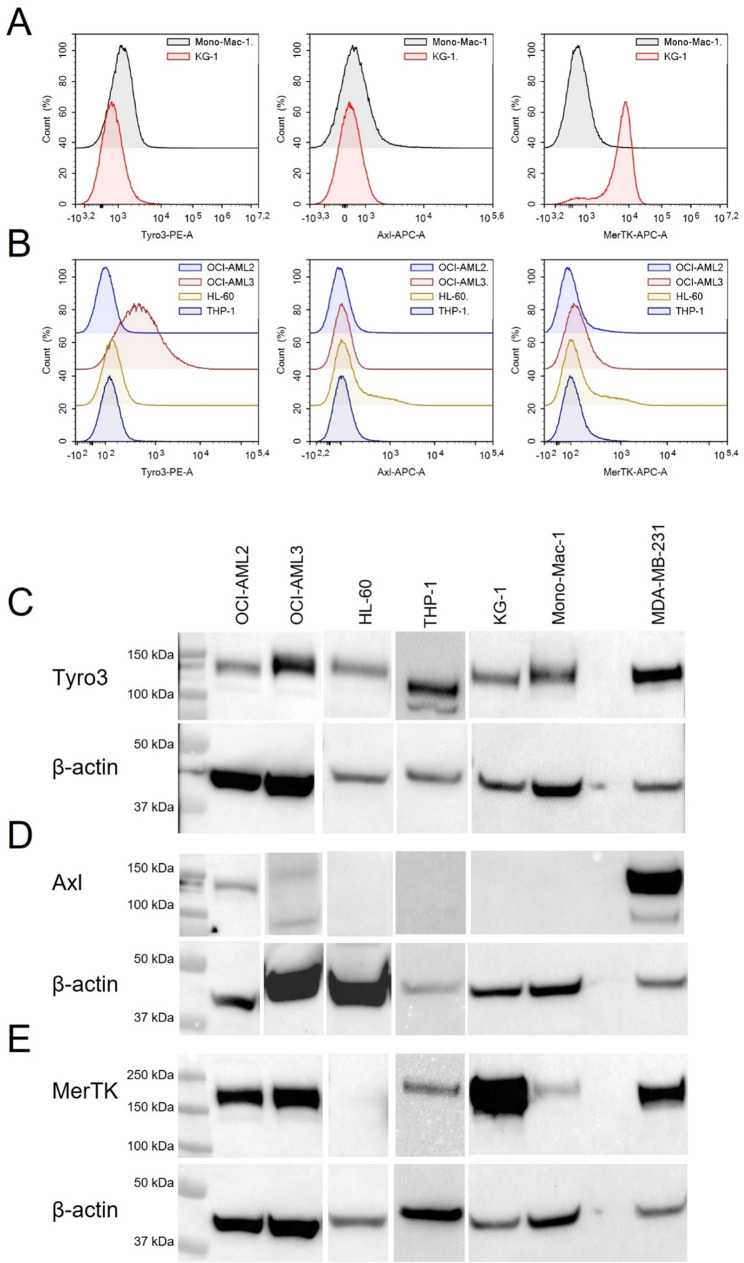
Variable surface and protein expression of Tyro3, AXL and MerTK receptors on AML cell lines. (**A**-**B**) AML cell lines Mono-Mac-1, KG-1, OCI-AML2, OCI-AML3, HL-60 and THP-1 were subjected to extracellular staining of Tyro3, AXL and MerTK receptors. Samples were acquired on a (**A**) NovoCyte Quanteon flow cytometer or (**B**) a BD FACS Canto flow cytometer. (**C**-**E**) Protein detection of (**C**) Tyro3, (**D**) AXL and (**E**) MerTK receptors by Western blotting. To enhance clarity, panels 1 C-E are compiled from different Western blot runs. β-actin expression was used as a loading control, and breast cancer cell line MDA-MB-231 was included as a positive control in a separate lane on every blot (exemplified in the right most column)

We then analyzed TAM receptor expression in myeloblasts from patient bone marrow aspirates (*n* = 25) and after short-term culture (*n* = 3). High variability in cell number and viability (Fig. 2A-C) reflects disease biology. MerTK expression was highest (mean DMFI 589.0 ± 100.4), Tyro3 was moderate (mean DMFI 235.1 ± 43.12), and AXL was low or absent (mean DMFI 43.4 ± 22.0). AXL was undetectable (DMFI ≤ 68) in 20/25 patients (Fig. 2D). After culturing for 48 h, AXL expression remained very low, though DMFI was higher compared to freshly thawed samples (Supplementary Figure S2). The 48-hour culture time was selected to ensure the cells were viable and stable, avoiding the stress and apoptosis commonly observed immediately after thawing.

Taken together, our study shows unexpectedly low AXL expression in bone marrow myeloblasts from 25 patients with myeloid neoplasms.

Previous studies have identified AXL overexpression in myeloid leukemias, linking it to the efficacy of AXL inhibitors. Overexpression is also observed in various solid tumors [4] often serving as a negative prognostic factor. However, clinical responses to AXL inhibitors extend beyond AXL-positive tumors. For instance, a cohort of 27 unselected relapsed or refractory AML patients, unsuitable for intensive therapy, treated with Bemcentinib (AXL inhibitor) and low-dose Cytarabine demonstrated promising survival results, with an overall survival (OS) of 13.3 months compared to 4.5 months in historical controls [8]. Additionally, AXL-negative non-small cell lung cancer (NSCLC) patients also responded to treatment, although response rate was lower than AXL-positive patients by immunohistochemistry [9].

**Fig. 2:**
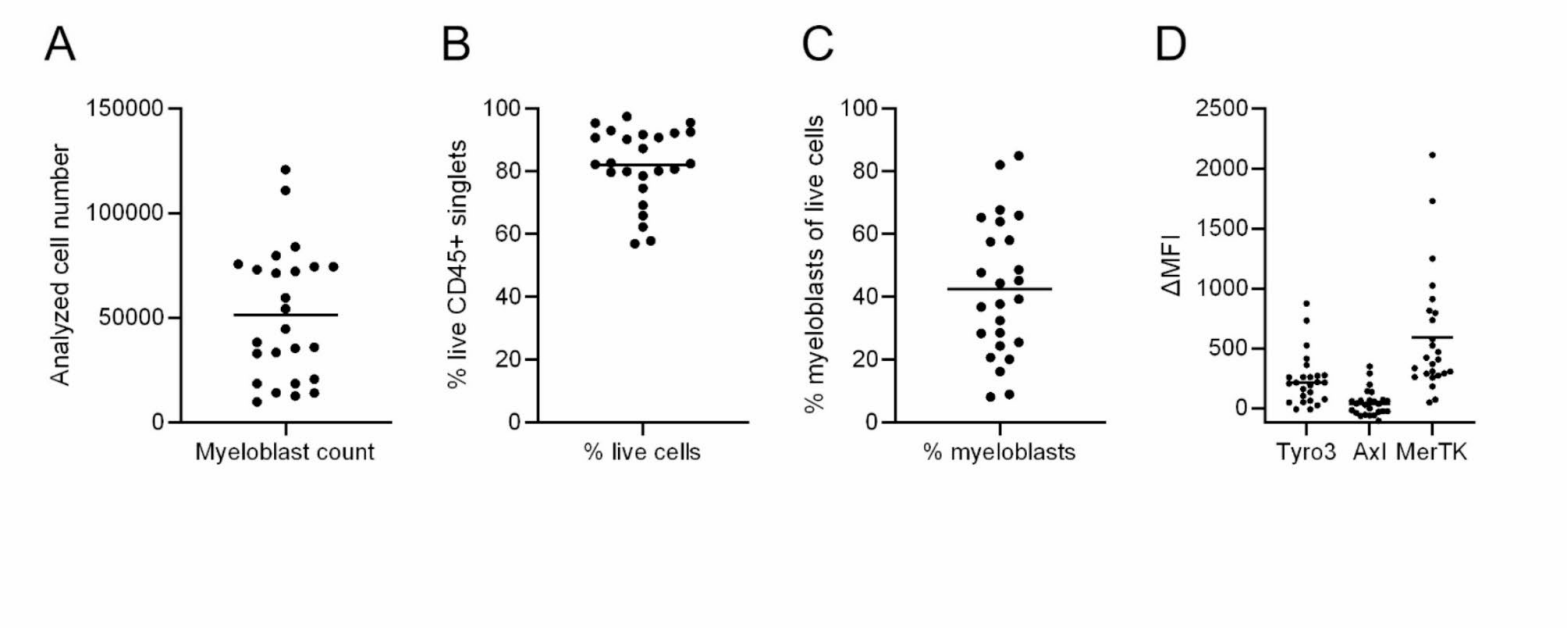
Assessment of Tyro3, AXL and MerTK expression on AML bone marrow patient samples. Surface expression of Tyro3, AXL and MerTK on myeloblasts of freshly thawed patient AML bone marrow samples was analyzed by flow cytometry. Gating was guided by fluorescence-minus-one controls. (**A**) Absolute number of analyzed myeloblast numbers per samples, as calculated by the absolute count function of the NovoExpress software. (**B**) Percentage of live cells was determined based on CD45 + singlets. (**C**) Percentage of myeloblasts was assessed out of live cells, defined as CD117+, with varying expression of CD34 + and CD13+. If possible, myeloblasts were gated as CD117 + and CD13+, only if there were no CD13 + cells in the sample, gating was done on CD117 + cells. In one single case, a sample was CD117- but CD13 + and defined as myeloblasts. (**D**) Expression of Tyro3, AXL and MerTK receptors is shown as the difference in median fluorescence intensity (ΔMFI) between the full stain and the fluorescence-minus-one (FMO) control. *n* = 25

These findings suggest that therapeutic efficacy may not solely arise from direct effects on tumor cells but may also involve alternative mechanisms. Immunoregulatory effects, independent of AXL receptor expression on tumor cells, may play a significant role [1, 10].

AXL signaling is activated by the ligand GAS6 and membrane phosphatidylserine (PtdSer), which serves as an “eat me” signal for phagocytes such as macrophages. This mechanism allows for the immunologically silent removal of apoptotic cells by the innate immune system. Cancer cells can exploit this suppressive mechanism by expressing PtdSer, mimicking apoptosis and inhibiting strong anti-cancer immune responsess [11]. Thus, blocking AXL signaling may restore danger signaling in the tumor microenvironment, facilitating an inflammatory response against cancer [7, 11]. Indeed, activation of CD8 + T cells and plasma B cells has been observed in AML patients responding to AXL inhibition [8].

Mouse studies have shown that AXL knockout tumors exhibit significant differences compared to wild-type tumors. In mammary tumors, AXL suppresses MHCI expression and promotes cytokine secretion, enhancing macrophage activity [7]. Similar effects have been noted in pancreatic cancer, where AXL deficiency reduces tumor-associated macrophages while increasing CD3 + T cell and NK cell infiltration. Tirado-Gonzalez et al. demonstrated that the therapeutic benefits of AXL inhibition are immune-dependent, with macrophage AXL ablation triggering robust NK and T cell responses against AML, B-ALL, and nilotinib-resistant ALL in their mouse model [12].

Together, these data, along with our findings, suggest that AXL positivity is not a prerequisite for the therapeutic efficacy of AXL-inhibiting therapies. Given the promising therapeutic effects of AXL targeting, understanding the dominant mechanism of action in AML will be invaluable. This knowledge could facilitate the development of synergistic combination therapies beyond current strategies, ultimately improving the standard of care.

## Electronic supplementary material

Below is the link to the electronic supplementary material.

Supplementary Material 1

## Data Availability

The datasets generated during and/or analyzed during the current study are available from the corresponding author upon reasonable request.

## Abbreviations

ALL: Acute lymphoblastic leukemia
AML: Acute myeloid leukemia
CML: Chronic myeloid leukemia
CMML: Chronic myelomonocytic leukemia
EMT: Epithelial-mesenchymal transition
GAS6: Growth arrest-specific 6
MFI: Mean fluorescence intensity
NSCLC: Non-small cell lung cancer
OS: Overall survival
PtSer: Phosphatidylserine
