# Supplementary material for: AXL in myeloid malignancies – an elusive target?
Source: Biomark Res. 2024 Dec 18;12:158. doi: 10.1186/s40364-024-00704-8 (PMC11657231; doi:10.1186/s40364-024-00704-8)
Supplement: Supplementary file 1 — Supplementary Material 1 [file 40364_2024_704_MOESM1_ESM.pdf]

Supplementary Table 1: Patient characteristics of samples analyzed by flow cytometry.

**Age at diagnosis**		
	median (range)	71 (46-84)
**Sex**		
	female	12/25
**AML classification**		
	de novo	16/25
	secondary	8/25
	unknown	1/25
**ELN risk classification by genetics**		
	Favorable	1/25
	intermediate	17/25
	adverse	6/25
	not determined	1/25
**Sampling timepoint**		
	at diagnosis	22/25
	during treatment	2/25
	at relapse	1/25
**Leukocyte count (x10^9)**		
	median (range)	28,8 (1,1-169)
	missing information	4/25
**Blast percentage blood**		
	median (range)	64 (5-95)
**Blast percentage bone marrow**		
	median (range)	75 (18-97)

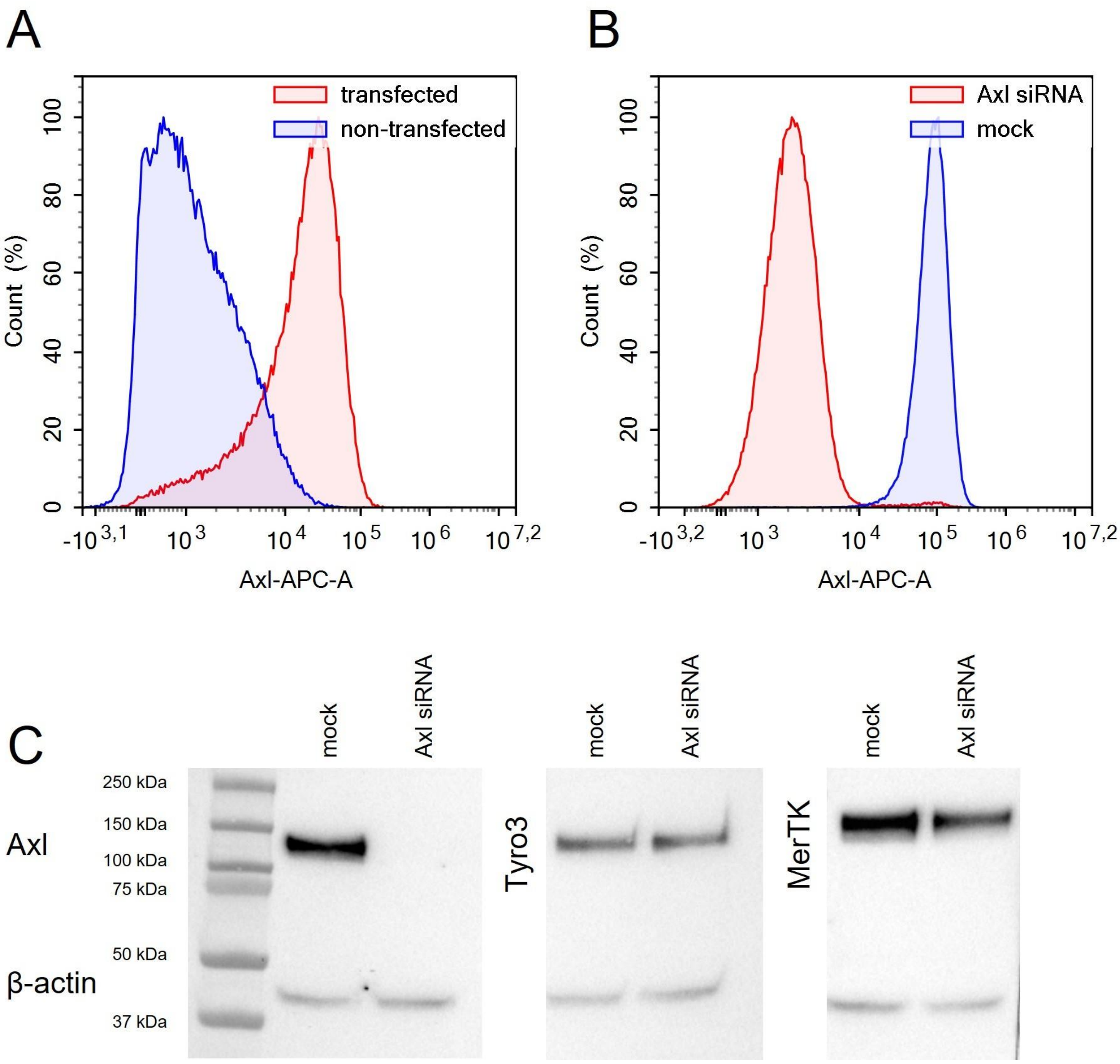
A
transfected
non-transfected
Count (%)
0
20
40
60
80
100
-10 3,1
10 3
10 4
10 5
10 6
10 7,2
Axl-APC-A
B
Axl siRNA
mock
Count (%)
-10 3,2
Axl-APC-A
C
mock
Axl siRNA
250 kDa
150 kDa
100 kDa
75 kDa
50 kDa
37 kDa
Axl
β-actin
Tyro3
MerTK

Supplementary Figure S1: Anti-AXL antibodies are AXL-specific. (A) Transfection of AXL-negative cell line THP-1 with plasmid DNA encoding an AXL construct led to AXL surface expression detected by flow cytometry after 24 hours. Transfected cells are depicted in red, non-transfected cells are depicted in blue. **(B)** siRNA knockdown of AXL expression in AXL-positive cell line MDA-MB-231 decreased AXL surface expression after 24 hours. Cells treated with AXL siRNA are shown in red, mock electroporated cells are shown in blue. **(C)** siRNA knockdown of AXL was also visible at a protein level as assessed by Western blotting. Protein expression of AXL, but not Tyro3 or MerTK, was reduced after electroporation.

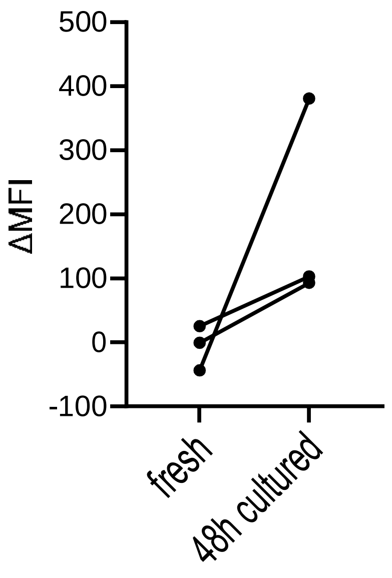

Supplementary Figure S2: Assessment of AXL expression on AML bone marrow patient samples after short-term culture. Surface expression of AXL on myeloblasts of patient AML bone marrow samples after 48h of short-term culture was analyzed by flow cytometry. Expression of AXL receptor is shown as the difference in median fluorescence intensity (ΔMFI) between the full stain and the fluorescence-minus-one (FMO) control. n=3

Material and Methods

Culture of cell lines

AML cell lines KG-1, OCI-AML2 and OCI-AML3 were kindly provided by Kirsten Grønbæk. OCI-AML2 and OCI-AML3 were cultured in Minimal Essential Medium (MEM) α (Gibco, Thermo Fisher Scientific, Waltham, MA, USA) + 20 % FCS at a concentration of 0.5-2x10^6 cells/ml. AML cell lines HL-60, THP-1, KG-1 and Mono-Mac1 as well as breast cancer cell line MDA-MB-231 were cultured in RPMI 1640 GlutaMAX-I™ medium (Gibco), supplied with 10 % FCS.

Western Blot

To determine TAM protein expression, cells were first lysed with RIPA Lysis and Extraction Buffer (Thermo Fisher Scientific), including Halt protease and phosphatase inhibitor cocktails (Thermo Fisher Scientific). Protein content of the lysates was determined with the Pierce BCA Protein Assay Kit (Thermo Fisher Scientific). 30-40 µg protein were loaded on 4-12 % Bolt Bis-Tris Plus Mini Protein gels (Invitrogen, Thermo Fisher Scientific, Waltham, MA, USA) and subsequently separated at 200 V for 20-25 min. Then, proteins were transferred to PVDF membranes with the iBlot 2 system (Invitrogen). For protein detection, the following primary antibodies were used: mouse anti-Axl (B-2, Santa Cruz Biotechnology, Dallas, TX, USA), rabbit anti-MerTK (D21F11 XP, Cell Signaling, Danvers, MA, USA), rabbit anti-Tyro3 (D38C6, Cell Signaling), mouse β-actin (C4, Santa Cruz Biotechnology). For protein visualization, HRP-linked secondary anti-mouse IgG (#7076, Cell Signaling) and anti-rabbit IgG (#7074, Cell Signaling) antibodies as well as SuperSignal West Pico PLUS Chemiluminescent Substrate (Thermo Fisher Scientific) were employed. Images were taken with a Bio-Rad ChemiDoc Molecular Imager.

Flow cytometry

AML cell lines were cultured for several passages before flow cytometry analysis. Patient material was thawed and analyzed immediately, or cultured for 48 h. For short-term culture, patient samples were thawed in RPMI and cultured in IMDM (Thermo Fisher Scientific) basal medium supplemented with 15 % BIT 9500 serum substitute (Stemcell Technologies, Vancouver, Canada) and 100 ng/ml stem cell factor (Merck Life Science, Darmstadt, Germany), 50 ng/ml FLT3 ligand (Merck Life Science), 20 ng/ml granulocyte colony-stimulating factor (Miltenyi, Bergisch Gladbach, Germany), 20 ng/ml IL-3 (Merck Life Science), 1 µM UM729 (Stemcell Technologies) and 500 nM SR1 (Stemcell Technologies) for 48 hours before flow cytometry analysis as described in Gottschlich et al..

First, cells were washed twice with PBS + 2 % FCS (FACS buffer), then incubated with an antibody mix at 4°C for 20-30 min before two additional washes with FACS buffer. Cells were resuspended in FACS buffer and acquired on the NovoCyte Quanteon (Agilent, Santa Clara, CA, USA) or on BD FACS Canto (BD Biosciences, Franklin Lakes, NJ, USA). Dead cells were excluded by staining with Live/Dead Near IR Fixable Stain (Thermo Fisher Scientific). In this study, the following antibodies were used: anti-Axl-APC (#108724), anti-MerTK-APC (#125518), anti-Tyro3-PE (#96201), all R&D systems (Minneapolis, MN, USA); anti-CD45-BB700 (HI30, BD Biosciences), anti-CD34-FITC (5G1, BioLegend, San Diego, CA, USA), anti-CD117-BV421 (YB5.B8, BD Biosciences), anti-CD13-BV605 (WM15, BioLegend). Gating was guided by fluorescence-minus-one controls. For analysis with the NovoExpress software (Agilent), live $CD45^+$ singlets were gated, then myeloblasts were defined as being $CD117^+$, with varying expression of $CD34^+$ and $CD13^+$. If possible, myeloblasts were gated as $CD117^+$ and $CD13^+$, only if there were no $CD13^+$ cells in the sample, gating was done on $CD117^+$ cells. In one single case, a sample was $CD117^-$ but $CD13^+$ and defined as myeloblasts.

siRNA electroporation

To knock down Axl protein expression, MDA-MB231 cells were electroporated with the following Silencer Select siRNAs (Thermo Fisher Scientific) targeting exons 16, 17 and 18: Axl siRNA 1:

GGAACUGCAUGCUGAAUGATT (sense), UCAUUCAGCAUGCAGUUCCTG (antisense); Axl siRNA 2: GGGUGGAGGUUAUCCUGAATT (sense), UUCAGGAUAACCUCCACCCTC (antisense); Axl siRNA 3: CAGCGAGAUUUAUGACUAUTT (sense), AUAGUCAUAAAUCUCGCUGTT (antisense). Before siRNA electroporation, MDA-MB231 cells were harvested by trypsinization and washed twice in cold Opti-MEM (Gibco). 0.25 nmol siRNA were added per $7x10^6$ cells; then the cells were transfected with one 2 ms, 500V unipolar pulse in a ECM830 square wave electroporation system (BTX, Holliston, MA, USA). After pulsing, cells were carefully transferred to a flask with pre-heated media and incubated for 24 hours before readout by flow cytometry and Western Blotting.

Transfection

Axl was overexpressed in Axl-negative cell line THP-1 through transfection of Axl plasmid DNA. The expression plasmid Axl Human Tagged ORF clone was purchased from Origene (Cat. no. RC206431, Rockville, MD, USA). To amplify the plasmid, competent *E. coli* were transformed with the plasmid and grown overnight on agar plates with kanamycin. Successfully transformed colonies were selected and expanded overnight in LB broth with kanamycin. Next, plasmid DNA (pDNA) was isolated with the PureLink HiPure Plasmid Filter Maxiprep kit (Invitrogen) according to the manufacturer's instruction. pDNA concentration was analyzed with a NanoDrop spectrophotometer (Thermo Fisher Scientific). pDNA was stored at -20 °C until use. THP-1 cells were transfected with the pDNA with the help of the TurboFect kit (Thermo Fisher Scientific). For that purpose, $0.5x10^6$ THP-1 cells were plated in a 24-well plate in R10. The next day, pDNA was diluted in serum-free media and incubated with the transfection mix for 20 min. Afterwards, 1 µg pDNA was added dropwise to each well. Flow cytometry readout was performed after 24 hours.

Patient material

The study was performed in accordance with the Declaration of Helsinki, and all 25 samples were collected following written informed consent. All patients were above 18 years of age. Capital

Region’s Ethics Committee approval H-20046888. Samples were stored in the Danish CancerBiobank.
